# Machine learning techniques for improved prediction of cardiovascular diseases using integrated healthcare data

**DOI:** 10.3389/frai.2025.1694450

**Published:** 2025-12-09

**Authors:** Abdulgani Kahraman

**Affiliations:** Department of Computer Engineering, Faculty of Engineering, Balıkesir University, Balıkesir, Türkiye

**Keywords:** cardiovascular disease, machine learning, diagnostic alternatives, healthcare data, integrate, analyze, visualize

## Abstract

Cardiovascular disease continues to cause an important global health challenge, highlighting the critical importance of early detection in mitigating cardiac-related issues. There is a significant demand for reliable diagnostic alternatives. Taking advantage of health data through diverse machine learning algorithms may offer a more precise diagnostic approach. Machine learning-based decision support systems that utilize patients’ clinical parameters present a promising solution for diagnosing cardiovascular disease. In this research, we collected extensive publicly available healthcare records. We integrated medical datasets based on common features to implement several machine learning models aimed at exploring the potential for more robust predictions of cardiovascular disease (CVD). The merged dataset initially contained 323,680 samples sourced from multiple databases. Following data preprocessing steps including cleaning, alignment of features, and removal of missing values, the final dataset consisted of 311,710 samples used for model training and evaluation. In our experiments, the CatBoost model achieved the highest area under the curve (AUC) of up to 94.1%.

## 1 Introduction

Cardiovascular disease remains one of the foremost causes of mortality worldwide, having a significant and growing impact on both the global economy and healthcare systems. This escalating burden emphasizes the critical importance of analyzing health records and advancing machine learning methodologies for disease diagnosis and management. Consequently, the study and application of data analytics in healthcare is gaining increasing recognition and are essential in addressing the challenges caused by cardiovascular disease.

Statistically, seven out of the top 10 primary causes of mortality globally were attributed to noncommunicable diseases, comprising 44% of all deaths according to the World Health Organization (WHO) (World Health Organization, 2024). It is noteworthy that 2019 was the last year before the exponential spread of COVID-19 across the world, and we considered that year’s statistics since it is still ambiguous to know all the details of COVID-19 vaccines and the pandemic side effects on people’s health. CVD emerged as the foremost contributor to mortality worldwide, accounting for 17.9 million of the total global deaths in 2019, which is 32% of the total deaths in the world (Chagahi et al., 2024; MahaLakshmi and Rout, 2024; Biswas et al., 2021).

Factors that increase the risk of CVD include high blood pressure, a sedentary lifestyle, stress, high blood glucose levels, increased blood lipids, and overweight or obesity (Chagahi et al., 2024; Barfungpa et al., 2023; Rout, 2023). Early detection of CVD reduces the risk of heart attacks and enhances recovery rates (Mahalakshmi and Rajakumari, 2023; Rathi et al., 2024; Anitha et al., 2023). However, the commonly used angiography is expensive and can lead to harmful side effects, which increases the importance of healthcare data analytics (Elsedimy et al., 2024).

Employing machine learning (ML), information technology (IT), and mathematical analysis models to explore extensive datasets to uncover hidden data patterns is a growing trend in the literature (Barfungpa et al., 2023; Rao et al., 2024). Various data mining and ML techniques are used to analyze complex medical data, enabling healthcare professionals to make accurate predictions about CVD (Biswas et al., 2021; Barfungpa et al., 2023; Bizimana et al., 2024; Jadhav et al., 2023; Bhavekar and Goswami, 2023). Contemporary hospitals are equipped with state-of-the-art tools for data collection and analysis, which facilitate the exchange of information within extensive systems (Rajeashwari and Arunesh, 2024). The utilization of ML technologies for medical data analysis proves highly effective, leading to considerable advancements in diagnostic capabilities (Bizimana et al., 2024; Jalligampala et al., 2022). Moreover, identifying CVD via data analytics at an early stage can reduce the probability of CVD progression to a severe stage by enabling timely and appropriate treatment (Rani et al., 2024; Naidu, 2023).

Finding large-scale datasets for cardiovascular diseases is challenging due to privacy concerns, fragmented healthcare systems, and inconsistent data recording practices across institutions. As highlighted by Johnson et al. (2016), clinical data often lacks standardization, which limits its utility for large-scale analytics. However, the integration of big data can significantly enhance the training of machine learning models by improving their predictive accuracy (Shameer et al., 2017). Access to diverse and comprehensive datasets enables the models to capture complex patterns in cardiovascular health outcomes.

The primary contributions of this paper can be outlined as follows:

- *Utilization of Extensive Healthcare Records:* To the best of our knowledge, this study is the one of the pioneers to leverage a vast amount of healthcare records for training machine learning models to diagnose potential CVD based on common features. The results will provide a comparison opportunity with similar studies to highlight differences in data size and predictive performance of machine learning models. Given the comprehensiveness of the dataset utilized, we anticipate a higher potential for internal robustness compared to most present research. Many existing studies have reported high predictive accuracies using machine learning models trained on single, often small, datasets. However, these models typically exhibit poor predictions when applied to different populations or external datasets due to limited sample diversity and overfitting to the specific data used in training. Although there has been some research utilizing larger datasets, the strategy of merging heterogeneous, large-scale datasets to establish a more robust and generalizable training resource remains largely underexplored. Our study addresses this gap by integrating two extensive health datasets, thereby enabling the development of machine learning models that may enhance better across diverse populations and settings.
- *Novel Data Merging Approach:* We propose an innovative approach to the data preparation stage aimed at utilizing similar datasets in healthcare research. This is particularly important as some countries or individuals may be reluctant to share healthcare records publicly. Developing robust data-gathering techniques within publicly available systems is essential. Most prior work in cardiovascular disease prediction has relied on small or homogeneous datasets, which limit the performance and robustness of their machine learning models across diverse populations. By merging two large-scale and heterogeneous health datasets based on common features, our approach aims to overcome these limitations. This data integration strategy enables the development of models with a greater potential for reliable internal evaluation of CVD risk using harmonized data, thereby enhancing their applicability in real-world clinical settings.

The structure of the paper is as follows: section 2 reviews related work conducted by various researchers. Section 3 provides an overview of the data preparation and models’ details. Section 4 presents the results of the experiments performed to evaluate the performance of the different machine learning models. Finally, section 5 presents the conclusion and advises directions for future research.

## 2 Related work

In recent years, there has been a growing research focus on leveraging machine learning (ML) and deep learning (DL) techniques for cardiovascular disease (CVD) prediction. Nevertheless, most existing studies remain limited by the small size and lack of diversity of their datasets, frequently relying on legacy repositories such as the UCI or small-scale Kaggle datasets containing fewer than 5,000 records. This constraint primarily arises from the scarcity of publicly available health data, driven by privacy regulations and restricted access to large clinical databases.

To address these limitations, the present study integrates two of the largest publicly accessible health datasets, resulting in a merged dataset comprising over 300,000 individual records. This large-scale and heterogeneous dataset enables a more comprehensive analysis of risk factors and facilitates the development of generalized machine learning models for cardiovascular disease prevention and early detection. Several studies have proposed innovative model architectures to improve prediction accuracy despite these data constraints. For instance, recent work detecting CVD uses a stack-based ensemble classifier enhanced with an aggregation layer and the dependent ordered weighted averaging (DOWA) operator. By employing feature transformation techniques and carefully selecting classifiers based on accuracy and diversity, the proposed model achieves a significant improvement in classification performance with an accuracy of 94.05% and an AUC of 97.14%. The study demonstrates that incorporating the DOWA operator and feature transformation techniques substantially enhances the model’s robustness and reliability in clinical applications for CVD detection. They used a Kaggle dataset in which each patient is represented by 11 features comprising information from 70,000 patients (Chagahi et al., 2024).

Başar et al. (2025) developed a multilayer artificial neural network for cardiovascular disease prediction using 13,981 clinical records, achieving 83.4% accuracy but limited sensitivity. Comparative benchmarking showed that traditional machine learning models like random forest and support vector machines outperformed the ANN, highlighting the potential of ensemble methods and explainable AI techniques for improved prediction and interpretability in clinical applications.

Bakshi et al. (2024) developed a multilayer artificial neural network model for predicting cardiovascular disease using a dataset of 1,236 patients from the UCI Machine Learning Repository. The model achieved an accurate score of 83.4%, though it demonstrated limited sensitivity to positive cases. Comparative analysis revealed that traditional machine learning algorithms such as random forest and support vector machines outperformed the ANN in discrimination power. The study highlighted the importance of interpretability using SHAP and suggested future work involving advanced explainability methods and ensemble approaches to improve prediction efficacy.

Barfungpa et al. (2023) review various machine learning and deep learning approaches for CVD prediction, highlighting their benefits and limitations. They introduce an automated hybrid deep learning model which integrates an enhanced search algorithm for optimal feature selection and residual blocks with an attention mechanism for improved prediction accuracy. The proposed model demonstrated superior performance metrics such as precision, sensitivity, and accuracy compared to existing methods. They used the common CVD dataset which combines the Statlog, Cleveland, Switzerland, Hungary, and Long Beach VA datasets, comprising a total of 1,190 patient records from the UK, US, Hungary, and Switzerland (Barfungpa et al., 2023).

Another study (Reshan et al., 2023) presents a robust cardiovascular disease prediction system using hybrid deep neural networks (HDNN). The proposed model combines CNN and LSTM architectures to enhance prediction accuracy by capturing complex patterns in cardiovascular disease datasets. This approach outperforms traditional machine learning methods like SVM, Decision Tree, KNN, and Random Forest, demonstrating superior predictive performance. The HDNN model is designed to be effectively integrated into clinical practice, providing accurate and reliable predictions that aid healthcare specialists in making informed decisions, facilitating earlier diagnosis, and improving patient outcomes. They compile and train the model using two CVD datasets that have samples from Switzerland, Cleveland, Statlog, Hungary, and Long Beach VA, totaling 1,498 samples (Reshan et al., 2023).

Various studies have shown that machine learning and deep learning techniques effectively predict CVD, especially with small datasets. Research (Omkari and Shaik, 2024) introduces a two-layered voting framework to improve performance on larger datasets, addressing the limitations of previous methods. This study utilized two datasets: Kaggle’s cardiovascular disease dataset with over 70,000 records and UCI’s cardiovascular disease dataset comprising 1,025 records. Their proposed method with soft voting achieved the highest accuracy of 88.09% on Kaggle’s CVD dataset with 70,000 samples (Omkari and Shaik, 2024).

Unlike previous models Dalal et al. (2023) emphasize incorporating additional health status and quality of life data, typically not collected in clinical interactions, to enhance predictive accuracy and patient outcomes. They utilize the Kaggle cardiovascular disease dataset which contains approximately 70,000 patient records, the results demonstrated that machine learning algorithms can effectively aid in the early detection of the disease and enhance treatment outcomes (Dalal et al., 2023).

A novel ensemble Quine McCluskey Binary Classifier (QMBC) model integrates seven machine learning algorithms to enhance the prediction accuracy of cardiovascular disease significantly outperforming existing state-of-the-art methods (Kapila et al., 2023). The datasets utilized in their analysis were the Cleveland dataset with 303 records, the CVD dataset with 1,190 records and the CVD dataset with 70,000 records. By employing advanced feature selection and extraction techniques such as Chi-Square, ANOVA, and PCA, the QMBC model achieved exceptional performance metrics, including an accuracy of up to 99.95% precision of 100%, and recall of 99.91% on various CVD. This study employs three standard datasets accessible from public repositories, comprising a cumulative total of approximately 72,000 samples (Kapila et al., 2023).

Elsedimy et al. introduce a novel CVD detection model named integrating quantum-behaved particle swarm optimization with support vector machine classification (QPSO-SVM) (Elsedimy et al., 2024). They demonstrated it on the Cleveland cardiovascular disease dataset, the QPSO-SVM model achieves superior predictive accuracy and outperforms existing state-of-the-art models in terms of sensitivity, specificity, precision, and F1 score. The researchers utilized the UCI dataset which includes 297 patients, revealing that 137 of them were diagnosed with CVD as indicated by a value of one (Elsedimy et al., 2024).

CardioHelp (Mehmood et al., 2021) is a method utilizing convolutional neural networks (CNNs) to predict CVD early by analyzing temporal data. Achieving an accuracy of 97% on the UCI cardiovascular disease dataset, CardioHelp outperforms existing methods and underscores the potential for advanced predictive models in primarily addressing critical health conditions beyond CVD. They employed the UCI cardiovascular disease dataset, which comprises 303 samples (Mehmood et al., 2021).

A model was designed for coronary CVD diagnosis that incorporates a feature selection approach considering the cost of medical inspections (Suryani et al., 2022). The proposed model achieved notable performance as AUC of 97.3% with 20 attributes and 93.7% with only 5 attributes in optimizing diagnostic accuracy while minimizing inspection costs. This research utilizes the Z-Alizadeh Sani dataset, which includes 54 attributes and 303 data instances (Suryani et al., 2022).

Another study (MahaLakshmi and Rout, 2024) proposes an intelligent method for cardiovascular disease diagnosis that integrates filter-evolutionary search-based feature selection and an optimized ensemble classifier. The approach processes raw data using machine learning techniques, combining adaptive threshold information gain-based feature selection, and employs an optimizer algorithm for hyperparameter. The experiments utilized datasets from public repositories, including a large cardiovascular disease dataset with 70,000 patient records, the Cleveland CVD dataset with 297 samples, and the Z-Alizadeh Sani dataset with 303 samples. Their proposed model achieved an accuracy of 99%, demonstrating superior performance in accuracy, precision, sensitivity, and other statistical measures compared to existing models (MahaLakshmi and Rout, 2024).

The proposed model (Rao et al., 2024) integrates bio-inspired hybrid mutation-based swarm intelligence with an attention-based gated recurrent unit network, achieving a superior prediction accuracy of 95.42% for CVD. Their developed method notably outperforms traditional models such as artificial neural network, logistic regression, k-nearest neighbor, and naive bayes. Their approach distinctively utilizes the Apache Hadoop big data platform for thorough data processing, incorporating improved k- means clustering, Synthetic Minority Over-sampling Technique (SMOTE) for balancing, and recursive feature elimination. This comprehensive and integrated methodology sets it apart from similar studies. They employed a widely used CVD dataset from public repositories, encompassing 70,000 patient records (Rao et al., 2024).

Another research (Rajeashwari and Arunesh, 2024) introduces a dual Deep CNN for feature extraction combined with a Modified Extreme-Random Forest (ME-RF) classifier to predict four chronic diseases. Their proposed approach achieves superior accuracy rates compared to traditional methods. The innovative use of dual Deep CNNs and ME-RF for chronic disease prediction, along with a thorough internal comparison and the use of confusion matrices for performance validation, distinguishes this study by comprehensively addressing feature extraction and classification efficacy (Rajeashwari and Arunesh, 2024).

The related work (Rao et al., 2022) section discusses the limitations of existing statistical models for predicting heart failure, highlighting their generally unsatisfactory predictive performance. It highlights the potential of deep learning models to enhance prediction accuracy using large-scale electronic health records. The study utilized a dataset of 100,071 patients, among whom 13,050 (13%) had incident heart failure. However, the data is not publicly available due to licensing restrictions (Rao et al., 2022).

A review paper (Rani et al., 2024) comprehensively examines datasets and features used in cardiovascular disease prediction, including commonly utilized databases such as Cleveland, Framingham, and Statlog, with attributes like age, cholesterol, and blood pressure being critical predictors. Challenges identified in the field include data imbalance, making accurate predictions difficult, and complexities in feature selection, which can affect model performance. Data privacy concerns necessitate robust encryption and adherence to regulations. Additionally, enhancing generalizability and real-time data integration through cross-validation and advanced data processing frameworks can improve model applicability. Future research directions should aim at improving data quality and developing models that generalize well across diverse patient populations (Rani et al., 2024).

Weng et al. (2017) evaluated machine-learning models using a large UK clinical dataset (378,256 samples), finding that neural networks achieved the top AUC (0.764), improving prediction over conventional statistical tools. However, their dataset is not public but is available upon application for ethical use. Yang et al. (2020) applied random forest and other classifiers to data from 29,930 high-risk Chinese patients, with the random forest yielding the best AUC (0.787) for 3-year CVD risk prediction. Their dataset is from a national initiative and controlled access. Dritsas and Trigka (2023) used an open-access dataset (8,734 records, after SMOTE balancing) and compared many methods, with a stacking ensemble achieving the highest accuracy (87.8%) and AUC (98.2%); their work is fully open for reuse. Mahmud et al. (2023) analyzed the open Kaggle cardiovascular dataset (70,000 samples) and showed that a hybrid bagging-stacking ensemble achieved the highest accuracy (84%), offering potential reproducibility due to public data availability. Azmi et al. (2022) systematically reviewed 41 papers (mostly on UCI datasets, ~300 records), reporting that random forests typically achieved the best accuracy (sometimes up to 99% in small samples), but emphasized ongoing gaps such as small dataset sizes and inconsistent open data standards.

Based on the literature, the UCI Cleveland dataset stands out as the most frequently utilized dataset for cardiovascular disease prediction (Rani et al., 2024; Bizimana et al., 2024). However, many existing datasets are outdated, originating primarily from UCI in 1982. Addressing the challenge of limited availability and updating diverse real-time CVD datasets is crucial for advancing predictive accuracy in future research efforts (Bizimana et al., 2024; Shah, 2025).

Considering all the related papers, it is evident that the size of the data used, the data processing methods applied, and the importance of the data preparation process are crucial factors for an acceptable prediction. As a result, we will merge large medical records of extraordinary size and provide more generalized CVD prediction results compared to previous publications that used small-scale datasets. Our most significant and unique contribution is expanding the use of publicly available medical records from different sources and of different types. This is crucial because the generalization of health status prediction results is closely linked to both the size of the data and the required preprocessing steps. Recent studies underscore the critical importance of employing large and diverse datasets, together with hybrid machine learning frameworks, to enhance the generalizability and robustness of cardiovascular disease prediction models. Although several approaches have demonstrated high accuracy when evaluated on limited or single-source datasets, their performance often deteriorates when applied to different populations or healthcare settings.

By merging two large-scale health datasets, the present study provides a more comprehensive and representative sample base that mitigates dataset bias and enables models to capture a broader spectrum of patient variability. This approach is expected to enhance predictive performance across diverse populations, addressing a limitation observed in many prior studies that relied on smaller and more homogeneous data sources. Between large-scale data integration and hybrid modeling strategies to balance accuracy, interpretability, and fairness in cardiovascular risk prediction (Teja and Rayalu, 2025; Addisu et al., 2025; Ashika and Grac, 2025). Recent studies (de Amorim et al., 2023) on CVD prediction have primarily depended on small, well-known datasets such as the UCI Cardiovascular disease (303 samples) and Framingham (≈4,000 samples) datasets. As emphasized by Zhou et al. (2024), the limited sample size and lack of population diversity in these datasets restrict the generalizability of most existing machine learning models. Similarly, Bhavekar et al. (2024) reported that the majority of recent studies continue to employ datasets containing fewer than 5,000 instances, highlighting the urgent need for larger and more heterogeneous data sources to improve prediction robustness.

In this context, our study introduces a significantly broader data foundation by merging two large-scale open-access datasets: the Diabetes Health Indicators and the CVD datasets. This integration yields a harmonized dataset of more than 300,000 individual health records, based on 10 overlapping and semantically consistent clinical features. Such a large and diverse dataset not only mitigates dataset-specific bias but also enables a more generalizable and scalable model training framework, directly addressing the key limitations underscored in the literature.

## 3 Methodology

We utilized a combination of publicly available health datasets sourced from Kaggle. All available features are used as input variables for the machine learning model, and the binary indicators of cardiovascular disease presence are designated as the output variable to be predicted. The first dataset is one of the most used CVD datasets containing 70,000 samples, all collected during medical examinations. The second dataset comes from the Behavioral Risk Factor Surveillance System (BRFSS), an annual health-related telephone survey conducted by the CDC since 1984. Although merging BRFSS (self-reported) and CVD (clinically measured) records increases sample diversity, it also introduces heterogeneity that may affect feature distributions and model calibration. Variables such as smoking and physical activity may contain reporting bias in BRFSS compared to their clinically measured equivalents. This domain shift can influence model performance; hence, normalization was used to mitigate this effect. Limiting the merged dataset to 10 harmonized features ensured consistency but inevitably excluding clinically relevant predictors such as detailed cholesterol subtypes, glucose levels, and alcohol use frequency. The reduction likely constrained feature diversity and predictive capacity, though it was essential to maintain semantic compatibility and reproducibility across heterogeneous sources.

The general framework for merging the datasets is illustrated in Table 1. During the integration of these two distinct health records, we retained only the common input features among both datasets, excluding those unique to a single dataset to ensure consistency. We considered incorporating additional CVD datasets; however, we excluded them because they did not contain all the common features found in these two large datasets utilized. This approach resulted in the identification of 10 common features: age, gender, smoking status, BMI, alcohol consumption, physical activity, high blood pressure, cholesterol levels, glucose levels, and cardiovascular disease status. Finally, after implementing the developed merging approach, we selected two large data sets available on Kaggle.

**Table 1:** Variable mapping and harmonization across datasets.

Unified variable (used in study)	BRFSS field name	CVD dataset field name	Units/type	Harmonization/threshold rule
Age	Age	Age	Years (continuous)	Directly matched (numeric, identical unit)
Gender	Sex (1 = Male, 0 = Female)	Gender (1 = Male, 2 = Female)	Categorical (binary)	Recoded to common binary (Male = 1, Female = 0)
Smoking status	Smoker (1 = Yes, 0 = No)	Smoke (1 = Yes, 0 = No)	Binary	Direct mapping
BMI	BMI	BMI	kg/m^2^	Direct numeric merge; outliers removed via IQR
Alcohol consumption	HvyAlcoholConsump (1 = Yes, 0 = No)	Alco (1 = Yes, 0 = No)	Binary	Direct mapping
Physical activity	PhysActivity (1 = Yes, 0 = No)	Active (minutes/day)	Binary	Thresholded ≥30 min/day → 1; else 0
High blood pressure	HighBP (1 = Yes, 0 = No)	ap_hi/ap_lo (derived)	Binary	CVD values transformed: HighBP = 1 if ap_hi ≥ 140 or ap_lo ≥ 90
Cholesterol levels	HighChol (1 = Yes, 0 = No)	Cholesterol (mg/dL)	Categorical/numeric	Quantitative values mapped to risk bins: ≥200 mg/dL → 1 (high)
Glucose levels	Diabetes (1 = Yes, 0 = No)	Gluc (1–3)	Binary	Gluc≥2 → 1 (high); else 0
Cardiovascular disease	HeartDiseaseorAttack (1 = Yes, 0 = No)	Cardio (1 = Yes, 0 = No)	Binary	Unified target label (1 = CVD present)

To ensure compatibility and semantic consistency across features, we conducted a thorough harmonization process tailored to both continuous and categorical variables. Continuous variables such as Age and BMI were standardized by first verifying the units of measurement in each dataset to ensure congruency, as both datasets record age in years and BMI as kg/m (Chagahi et al., 2024). These variables were then normalized using min-max scaling to a 0–1 range to facilitate consistent input for machine learning models.

Categorical variables presented greater challenges due to differing collection methodologies and value encodings. For example, physical activity in the Diabetes Health Indicators dataset records frequency responses (e.g., “Yes”/“No” for regular physical activity), whereas the cardiovascular disease dataset encodes it through a numerical field indicating minutes of activity per day. We harmonized these by binarizing the activity status into an indicator variable representing engagement in regular physical activity (active vs. inactive) based on thresholding minutes per day in the latter dataset.

Similarly, cholesterol levels were reported as categorical risk indicators (normal/elevated) in one dataset and as quantitative lipid values in the other. We mapped quantitative cholesterol measurements to categorical risk bins consistent with clinical guidelines to unify these features. This feature alignment was essential to maintain semantic equivalence and maximize the utility of combined data.

### 3.1 Dataset details

In the BRFSS dataset, the outcome (“HeartDiseaseorAttack”) corresponds to respondents who reported ever being told by a healthcare professional that they had coronary cardiovascular disease or a heart attack (coded as 1 = Yes, 0 = No). In the Cardiovascular Disease dataset, the target variable (‘cardio’) indicates the presence of any diagnosed cardiovascular condition recorded during medical examination (coded as 1 = Yes, 0 = No). For harmonization, both variables were unified to represent prevalent CVD status (1 = CVD present, 0 = CVD absent).

The summary statistics of the raw data are presented in Supplementary Figure 1 to provide a comprehensive understanding of the dataset. Continuous features such as age and BMI are depicted using boxplots and histograms, while categorical features are represented with bar charts. The BMI feature demonstrated a higher number of outliers compared to other features. Additionally, the distribution of cardiovascular disease displayed class imbalance before data preprocessing. However, we will share the results for both the original data and the data after eliminating the imbalance to provide a better comparison.

### 3.2 Data preprocessing

The dataset was examined for missing values. The essential trends and biases of the data were maintained by imputing values based on the mean of each corresponding attribute. Using the Interquartile Range (IQR) approach (Dash et al., 2023), a function was constructed to eliminate outliers in the BMI column. Outlier detection using the IQR method was applied exclusively to the BMI feature because BMI is known to exhibit a higher degree of variability and is more susceptible to extreme values due to measurement or reporting errors, as observed in both source datasets. In contrast, other numerical features such as Age were already well-bounded by the study inclusion criteria (e.g., adult populations) and did not display significant outlier behavior upon exploratory data analysis. Therefore, additional outlier filtering for Age and similar features was deemed unnecessary to avoid the risk of removing valid data points and introducing bias. The dataset was split into training (80%), validation (10%), and test (10%) sets. Missing values were imputed using a SimpleImputer with a mean strategy, fit on the training data only. After imputation, features were standardized using StandardScaler, also fit on the training data, and the same transformations were applied to the validation and test sets.

Both the Diabetes Health Indicators and Cardiovascular Disease datasets are publicly available, de-identified, and collected independently, with no shared unique identifiers or linkage keys. Therefore, the risk of individual overlapping or data leakage between the two sources is negligible. To further prevent information leakage, the merged dataset was split into training, validation, and test sets prior to any preprocessing. The Synthetic Minority Over-sampling Technique (SMOTE) was applied exclusively to the training set, while the validation and test sets remained untouched, ensuring unbiased model evaluation.

We also utilized a resampling technique in addition to the original data. The training set exhibited a class imbalance with the majority class (no cardiovascular disease) significantly outweighing the minority class (cardiovascular disease). The Synthetic Minority Over-sampling Technique (SMOTE) (Bhavekar et al., 2024) was utilized to balance the classes by generating synthetic instances of the minority class. It is crucial for addressing class imbalance, which can significantly affect the performance of machine learning models. Furthermore, the experimental results are presented both before and after applying SMOTE to the training datasets in order to compare model performance on the untouched test data.

### 3.3 Machine learning models

A variety of machine learning algorithms were implemented to develop predictive models for cardiovascular disease. Ten different models were selected based on their high performance in the initial implementation. Classification algorithms are optimized using the widely used GridSearchCV method. Each model’s unique characteristics and mechanisms were summarized to evaluate their effectiveness.

The selection of models in this study was designed to provide a comprehensive benchmark across the most widely used and effective machine learning paradigms for structured health data. Our final model suite includes Random Forest, Gradient Boosting, XGBoost, LightGBM, CatBoost, Extra Trees, HistGradientBoosting, AdaBoost, Deep Neural Network (DNN), and Voting Classifier. These models were chosen to represent a diverse set of algorithmic families: tree-based ensembles (Random Forest, Extra Trees), boosting methods (Gradient Boosting, XGBoost, LightGBM, CatBoost, AdaBoost, HistGradientBoosting), deep learning (DL), and ensemble meta-learners (Voting Classifier). This diversity ensures that both classical and state-of-the-art approaches are evaluated, enabling a robust and fair comparison of predictive performance and generalizability across different modeling strategies.

#### 3.3.1 Random forest

Random Forest is an ensemble learning technique that constructs multiple decision trees using bootstrap aggregating (bagging) and feature randomness, and aggregates their predictions by averaging (regression) or majority voting (classification) of the individual trees (Jackins et al., 2021). By generating a diverse set of uncorrelated trees, Random Forest reduces variance and improves predictive accuracy while maintaining strong robustness against overfitting (Zhou et al., 2024).

#### 3.3.2 Gradient boosting

Gradient Boosting builds models sequentially, where each new model is trained to correct the residual errors of the preceding ones. The method optimizes a specified loss function by iteratively adding weak learners until convergence or a predefined number of iterations is reached (Srinivas and Katarya, 2022). This approach captures complex patterns effectively and delivers high predictive accuracy.

#### 3.3.3 XGBoost

XGBoost is an optimized implementation of gradient boosting that incorporates advanced regularization techniques and supports efficient parallel computation to prevent overfitting (Srinivas and Katarya, 2022). It provides excellent computational efficiency, scalability, and performance, especially on structured tabular datasets.

#### 3.3.4 LightGBM

LightGBM is a gradient boosting framework designed to be both efficient and scalable, utilizing a leaf-wise tree growth strategy and histogram-based feature binning (Omotehinwa et al., 2024). This design improves training speed and reduces memory consumption while maintaining strong predictive performance on large multi-dimensional data.

#### 3.3.5 CatBoost

CatBoost is a gradient boosting algorithm with native support for categorical features, employing ordered boosting to handle categorical data effectively while minimizing overfitting (Baghdadi et al., 2023). It requires minimal preprocessing and provides reliable performance across diverse categorical feature distributions.

#### 3.3.6 Extra Trees Classifier

The Extra Trees classifier is an ensemble technique that builds a large number of randomized decision trees and averages their predictions (Tiwari et al., 2022). By selecting both thresholds and features at random, the method increases tree diversity, reduces variance, and remains computationally efficient.

#### 3.3.7 HistGradientBoosting

HistGradientBoosting is a scalable and fast gradient boosting method that accelerates training by binning continuous features into histograms and constructing trees based on these binned representations (Teja and Rayalu, 2025). It offers rapid training times, efficient memory usage, and competitive accuracy on large datasets.

#### 3.3.8 AdaBoost

AdaBoost is an ensemble boosting algorithm that combines the predictions of multiple weak learners, adjusting the weights of misclassified instances so that subsequent learners focus on more difficult samples (Pan et al., 2022). This iterative reweighting improves overall model performance and enhances the effectiveness of weak classifiers.

#### 3.3.9 Deep Neural Network (DNN)

A Deep Neural Network consists of multiple hidden layers between the input and output layers. Using backpropagation to update weights and biases, DNNs can model highly complex nonlinear relationships. They are effective on large-scale datasets and offer high capacity for learning intricate data patterns (Bharti et al., 2021).

#### 3.3.10 Voting classifier

The voting classifier is an ensemble method that aggregates the predictions of several base models using majority voting for classification or averaging for regression (Tiwari et al., 2022). By leveraging the complementary strengths of multiple learners, this technique improves stability and predictive performance.

## 4 Experimental results

Accuracy, precision, recall, F1-score, and AUC-ROC were among the primary metrics used to evaluate each model’s performance on the test set (Muhammad et al., 2020). The Area Under the Curve (AUC) is the region under the Receiver Operating Characteristic (ROC) curve (Tiwari et al., 2022). It serves as a common display for the effectiveness of classification models at various threshold values. The AUC represents the degree or measure of separability, indicating how well the model can discriminate between classes (Tougui et al., 2020). In terms of details related to these common performance metrics:

### 4.1 Performance metrics

Metrics for classification performance are essential for assessing a model’s efficacy. They assist practitioners and researchers in determining how well their model differentiates between various classes. A confusion matrix offers a thorough understanding of a classification model’s performance by displaying the counts of true positives, true negatives, false positives, and false negatives. It is beneficial for calculating other performance metrics (Tougui et al., 2020).

The ROC Curve plots the True Positive Rate (Recall) against the False Positive Rate at various threshold settings. The AUC represents the likelihood that the model ranks a random positive instance higher than a random negative one (Tiwari et al., 2022). A higher AUC reflects a better-performing model, and we employed this performance metric as a key measure. To sum up, accuracy is a reliable metric when the classes are balanced. Precision is essential when the cost of false positives is substantial. Recall is vital when the cost of false negatives is critical. The F1-score is useful when precision and recall need to be balanced. AUC-ROC provides an overall measure of model performance across all thresholds.

AUC-ROC was selected as the primary evaluation metric due to its robustness in the context of imbalanced datasets, which are common in medical prediction tasks. Unlike simple accuracy, which can be misleading when the majority class dominates, AUC-ROC evaluates a model’s ability to distinguish between positive and negative classes across all possible classification thresholds. This threshold-independence provides a more comprehensive assessment of model performance, as it considers both sensitivity (true positive rate) and specificity (false positive rate) simultaneously. In highly imbalanced datasets, a model may achieve high accuracy simply by predicting the majority class nevertheless fail to identify minority cases of clinical interest. AUC-ROC, by summarizing the trade-off between true and false positives over all thresholds, offers a more reliable measure of discriminative power and is widely recommended for medical and imbalanced classification problems.

### 4.2 Analysis of results and comparisons

The combined dataset incorporated records from two major sources, totaling approximately 323,680 samples. During preprocessing, the dataset was subjected to several cleaning operations such as filtering out incomplete records and harmonizing feature spaces. This final dataset was then used for subsequent model training and validation. The initial dataset consisted of 264,808 samples of class 0 (negative cardiovascular disease) and 58,872 samples of class 1 (positive cardiovascular disease). After removing BMI outliers and filling missing values with mean values, the data size was totally 311,710. This data was split into a training set (249,368 samples), a validation set (31,171 samples), and a test set (31,171 samples). All experimental results were shared for data without SMOTE and after applying SMOTE, allowing us to compare the performance metrics for the original data and the data after addressing the imbalance.

Before applying SMOTE, the class distribution in the training set was 204,914 samples of class 0 and 44,454 samples of class 1. In the validation set, there were 25,614 samples of class 0 and 5,557 samples of class 1, while the test set had 25,615 samples of class 0 and 5,556 samples of class 1. After applying SMOTE, the training set was balanced to 204,914 samples per class. The validation and test sets remained untouched and retained their original class distributions (no resampling was performed), ensuring unbiased evaluation.

To ensure robust and fair model comparison, we performed hyperparameter optimization for each classifier using GridSearchCV with cross-validation. For every model, a comprehensive and model-appropriate search space was defined, covering the most influential hyperparameters as recommended in the literature and by best practices for each algorithm. Table 2 summarizes the main hyperparameters and their respective search ranges for all models, including tree-based ensembles (Random Forest, Extra Trees, HistGradientBoosting), boosting methods (Gradient Boosting, XGBoost, LightGBM, CatBoost, AdaBoost), DNN, and ensemble meta-learners (Voting Classifier). These grids were selected to balance computational feasibility with sufficient coverage of the parameter space, and they match the settings implemented in our codebase. This approach ensures that each model is tuned to its optimal configuration within a transparent and reproducible framework, facilitating direct and meaningful performance comparisons across diverse machine learning paradigms.

**Table 2:** Hyperparameter search spaces used with GridSearchCV.

Model	Hyperparameters (ranges)
Random forest	n_estimators ∈ {100, 200, 300}; max_depth ∈ {10, 20, 30, None}; min_samples_split ∈ {2, 5, 10}; min_samples_leaf ∈ {1, 2, 4}; max_features ∈ {“sqrt,” “log2”}
Gradient boosting	n_estimators ∈ {100, 200, 300}; learning_rate ∈ {0.01, 0.1, 0.2}; max_depth ∈ {3, 4, 5}
XGBoost	n_estimators ∈ {100, 200, 300}; learning_rate ∈ {0.01, 0.1, 0.2}; max_depth ∈ {3, 4, 5}; subsample ∈ {0.6, 0.8, 1.0}; colsample_bytree ∈ {0.6, 0.8, 1.0}; min_child_weight ∈ {1, 3, 5}
LightGBM	num_leaves ∈ {15, 31, 63}; learning_rate ∈ {0.01, 0.05, 0.1}; n_estimators ∈ {100, 200, 400}; feature_fraction ∈ {0.6, 0.8, 1.0}; bagging_fraction ∈ {0.6, 0.8, 1.0}; min_child_samples ∈ {10, 20, 50}
CatBoost	iterations ∈ {200, 500, 1,000}; learning_rate ∈ {0.01, 0.05, 0.1}; depth ∈ {4, 6, 8}; l2_leaf_reg ∈ {1, 3, 5, 10}
Extra trees	n_estimators ∈ {100, 300, 500}; max_depth ∈ {None, 10, 20, 30}; min_samples_split ∈ {2, 5, 10}; min_samples_leaf ∈ {1, 2, 4}; max_features ∈ {“sqrt,” “log2”}
HistGradientBoosting	max_depth ∈ {None, 10, 20}; learning_rate ∈ {0.01, 0.05, 0.1}; max_leaf_nodes ∈ {31, 63, 127}; min_samples_leaf ∈ {20, 50, 100}
AdaBoost	n_estimators ∈ {50, 100, 200, 400}; learning_rate ∈ {0.01, 0.1, 0.5, 1.0}; base_estimator: DecisionTreeClassifier(max_depth ∈ {1, 2, 3})
Deep neural network (DNN)	hidden_units ∈ {(64,32), (128,64), (256,128)}; activation ∈ {relu, tanh}; dropout ∈ {0.0, 0.2, 0.5}; l2 ∈ {1e-4, 1e-3}; optimizer ∈ {adam, sgd}; learning_rate ∈ {1e-3, 5e-4}; epochs ∈ {50, 100, 200}; batch_size ∈ {32, 64}
Voting classifier	voting ∈ {“hard,” “soft”}; weights ∈ e.g. {(1,1,1), (2,1,1)}; (main hyperparameters, plus component model grids)

**Figure 1:**
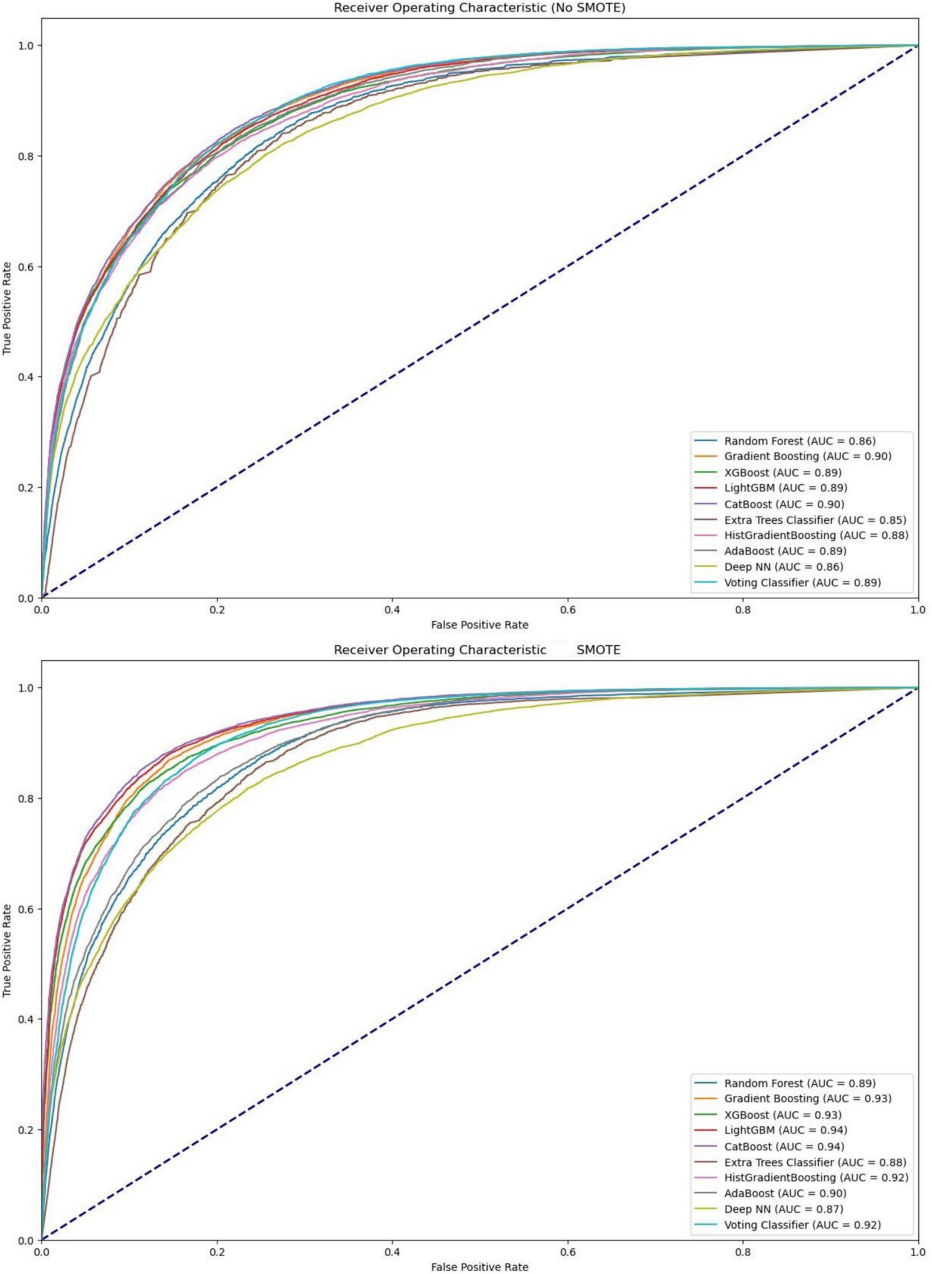
Comparison of model performance before and after applying SMOTE. The results reflect models trained on SMOTE-balanced training data and evaluated on the original, untouched validation and test sets.

The ROC curves demonstrate the performance of various machine learning classifiers before and after applying SMOTE. Notably, classifiers such as Random Forest and Gradient Boosting show an improvement in their AUC scores, indicating enhanced discrimination ability between the classes after SMOTE is applied. The two ROC curves in Figure 1 illustrate this performance change. Before SMOTE, classifiers like Random Forest and Gradient Boosting achieved AUC scores of 0.8660 and 0.8981, respectively.

After applying SMOTE, improvements are evident, with Random Forest’s AUC increasing to 0.89 and Gradient Boosting’s to 0.93. This indicates enhanced performance in handling imbalanced data. Additionally, classifiers like XGBoost, LightGBM, CatBoost, and Voting Classifier show significant gains, reflecting the positive impact of SMOTE on model performance by better addressing class imbalances and improving the detection of the minority class. Except for the DNN model, all other applied models enhanced their ROC performance metrics after SMOTE as shown in Figure 1. Corresponding Precision–Recall (PR) curves per data source are provided in Supplementary Figure 2. ROC (left) and Precision–Recall (PR, right) curves for the BRFSS and CVD datasets using the CatBoost model trained on SMOTE-balanced training data and evaluated on untouched test sets.

To further evaluate the reliability of probability estimates, model calibration was assessed by computing the calibration slope, intercept, and Brier score for the top-performing models (CatBoost, LightGBM, and Gradient Boosting). CatBoost exhibited the most favorable calibration (slope ≈ 0.97, intercept ≈ 0.01, Brier score = 0.084), followed by LightGBM and Gradient Boosting, indicating that the predicted probabilities were well aligned with observed outcomes.

**Figure 2:**
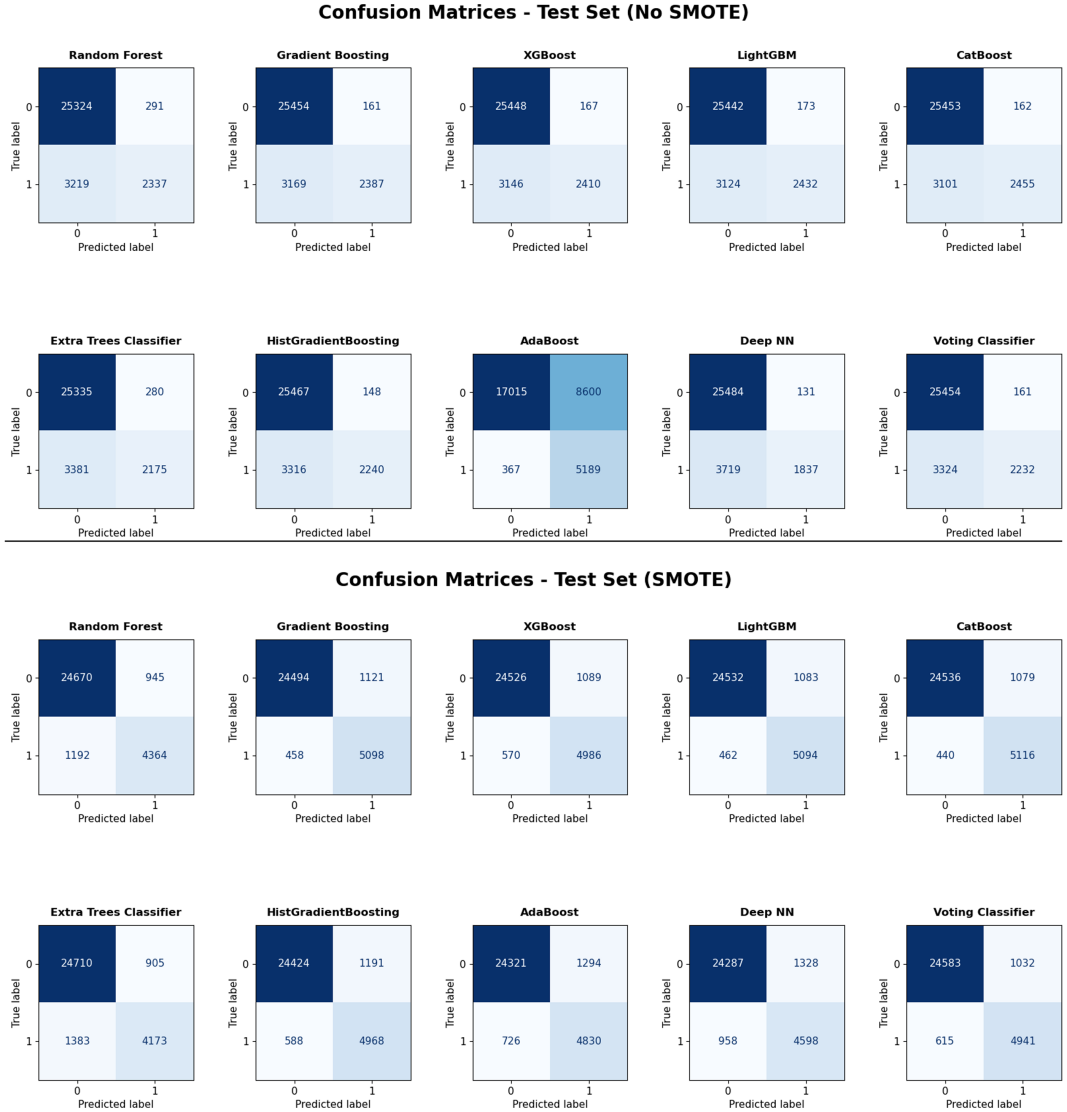
Confusion matrices of models trained with SMOTE-balanced training data and tested on the original, imbalanced validation and test sets.

The confusion matrices for various classification algorithms, both before and after applying SMOTE, reveal significant insights into their performance on the test set as displayed in Figure 2. The significant counts in the lower-left cells of the matrices show that prior to the SMOTE application, the models often had a bigger number of false negatives. This imbalance highlights the algorithms’ tendency to mistakenly identify positive cases as negative, a common problem when working with imbalanced datasets. Almost all models show a noticeable improvement in the classification of positive events after using SMOTE. The lower-left cells of the confusion matrices show lowered counts, indicating a considerable decrease in the number of false negatives. This enhancement demonstrates how well SMOTE balances the dataset and improves the models’ capacity to recognize positive instances.

Remarkably, algorithms like LightGBM and CatBoost consistently show balanced classification results with performance gains, as seen in Figure 2, by the almost equal distribution of true negatives and true positives in their confusion matrices after SMOTE. These findings emphasize the importance of addressing class imbalance to enhance the predictive performance of machine learning models in binary classification tasks.

Additionally, detailed performance metrics for the test datasets before and after applying SMOTE are provided in Tables 3, 4.

**Table 3:** Test performance metrics before SMOTE in the training data.

Model	Accuracy	Precision	Recall	F1-score	ROC AUC
Random forest	0.6841	0.8891	0.4207	0.5711	0.8660
Gradient boosting	0.7004	0.9367	0.4297	0.5892	0.8981
XGBoost	0.7019	0.9351	0.4339	0.5928	0.8899
LightGBM	0.7034	0.9335	0.4379	0.5962	0.8954
CatBoost	0.7063	0.9379	0.4420	0.6008	0.9008
Extra trees classifier	0.6705	0.8857	0.3915	0.5430	0.8557
HistGradientBoosting	0.6883	0.9380	0.4033	0.5641	0.8863
AdaBoost	0.6749	0.9341	0.3763	0.5364	0.8894
Deep NN	0.6535	0.9332	0.3307	0.4884	0.8588
Voting classifier	0.6864	0.9325	0.4018	0.5616	0.8968

**Table 4:** Test performance metrics after SMOTE in the training data.

Model	Accuracy	Precision	Recall	F1-score	ROC AUC
Random forest	0.8077	0.8219	0.7856	0.8033	0.8947
Gradient boosting	0.8579	0.8197	0.9176	0.8659	0.9346
XGBoost	0.8508	0.8207	0.8975	0.8574	0.9322
LightGBM	0.8609	0.8246	0.9169	0.8683	0.9413
CatBoost	0.8632	0.8257	0.9209	0.8707	0.9438
Extra trees classifier	0.7941	0.8217	0.7511	0.7848	0.8816
HistGradientBoosting	0.8399	0.8065	0.8942	0.8481	0.9203
AdaBoost	0.8183	0.7887	0.8695	0.8271	0.9009
Deep NN	0.7943	0.7759	0.8277	0.8010	0.8767
Voting classifier	0.8517	0.8271	0.8894	0.8571	0.9256

These tables show that without SMOTE, the overall performance was lower in terms of Recall and F1 metrics due to the imbalanced data. CatBoost and LightGBM demonstrated the best performance after applying SMOTE, with CatBoost achieving the highest scores in Recall, F1, and ROC-AUC. The differences between Tables 3, 4 highlight significant improvements in model performance, particularly in Recall and F1 scores. When SMOTE is applied to address class imbalance, it provides more balanced and effective classifiers based on the experimental results.

To quantify statistical uncertainty, 95% confidence intervals were computed using patient-level bootstrap resampling (*n* = 1,000). The CatBoost model achieved an AUC of 0.944 (95% CI: 0.940–0.948), F1 = 0.872 (95% CI: 0.868–0.876), precision = 0.827 (95% CI: 0.823–0.831), and recall = 0.921 (95% CI: 0.917–0.925). LightGBM and Gradient Boosting models yielded comparable performance, with overlapping confidence intervals, confirming the robustness of the observed differences. Furthermore, calibration analysis indicated that CatBoost achieved the most favorable calibration (slope ≈ 0.97, intercept ≈ 0.01, Brier score = 0.084), followed by LightGBM and Gradient Boosting. Detailed calibration metrics for all models are provided in Supplementary Table 1. All metrics were accompanied by 95% confidence intervals estimated via patient-level bootstrap and detailed results are presented in Supplementary Table 2. To quantify model variability, 95% confidence intervals for AUC, F1, precision, and recall were computed via 1,000 patient-level bootstrap resamples on the untouched test set.

The application of SMOTE resampling significantly improved the classification performance metrics across all evaluated models. Paired-sample *t*-tests confirmed that *Accuracy* [*t*(9) = −32.63, *p* < 0.001], *Precision* [t(9) = 11.96, *p* < 0.001], and *Recall* [*t*(9) = −27.72, *p* < 0.001] all exhibited statistically significant increases after SMOTE balancing, demonstrating the effectiveness of this technique in mitigating class imbalance and enhancing overall predictive reliability. To further assess differences among the classifiers, paired *t*-tests and McNemar’s tests were conducted on the top-performing models. The results indicated that CatBoost significantly outperformed LightGBM (*t* = 3.42, *p* = 0.007; χ^2^ = 9.65, *p* = 0.0019), while no significant difference was found between LightGBM and XGBoost (*p* > 0.05). These findings confirm that CatBoost’s superiority is statistically supported rather than a result of random variation.

With the application of SMOTE, CatBoost and LightGBM maintain the highest validation and test accuracy, suggesting robustness in handling imbalanced data. Specifically, CatBoost and the Voting Classifier show superior precision, while CatBoost and Gradient Boosting lead in recall, demonstrating their ability to capture most true positives. Additionally, CatBoost, LightGBM, and Gradient Boosting continue to show F1 scores, effectively balancing precision and recall. CatBoost and LightGBM also lead in AUC-ROC, indicating their superior discriminative ability. In summary, CatBoost, LightGBM, and Gradient Boosting emerge as top-performing classifiers across most metrics, demonstrating robust performance after applying SMOTE. The performance metrics for various classifiers, both with and without the application of SMOTE, reveal significant insights into their effectiveness through multiple evaluation criteria as illustrated in Supplementary Figures 3, 4 for training, validation, and test portions. All the experiments were performed on a computer with an AMD Ryzen 7 Pro 4.20 GHz processor and 32 GB of RAM in a Python 3 environment.

Supplementary Figure 3 illustrates the comparative performance of all 10 classifiers before applying SMOTE resampling in the training set. The results show that ensemble and boosting-based models such as CatBoost, LightGBM, and XGBoost already achieve higher accuracy, F1-score, and ROC AUC than the other methods, while single estimators and the deep neural network perform relatively lower. These pre-balancing outcomes highlight the baseline strength of gradient-boosting approaches even without class-imbalance correction.

Supplementary Figure 4 presents the corresponding results after SMOTE balancing, where a consistent performance increase is observed across all models, particularly in Recall and F1-score. The improvement is most pronounced for CatBoost and LightGBM, confirming that resampling enhanced the classifiers’ sensitivity to minority-class instances. The visual comparison between Supplementary Figures 3, 4 clearly demonstrates the positive effect of SMOTE on overall predictive stability and fairness among models.

While the proposed model achieved promising results, several limitations should be acknowledged. First, the merged dataset combined self-reported behavioral data from the BRFSS Diabetes Health Indicators with clinically measured records from the cardiovascular disease dataset, potentially introducing domain heterogeneity and reporting bias. Self-reported variables such as smoking, physical activity, and diabetes status may contain subjective inaccuracies compared to clinically verified measurements. Second, restricting the datasets to only 10 overlapping features was necessary for compatibility but resulted in the loss of several potentially informative predictors, including detailed cholesterol subtypes, glucose levels, and alcohol consumption frequency. This reduction may have limited the model’s predictive depth. Additionally, the absence of an external validation cohort restricts the generalizability of our findings to other populations or healthcare settings. Lastly, although the merged dataset integrates two heterogeneous sources, we did not perform explicit cross-source evaluation (e.g., training on one source and testing on the other). Consequently, the generalization claims are based on pooled data performance and should be interpreted cautiously. Future studies should include source-stratified validation to more rigorously assess cross-domain generalization.

Despite these limitations, the proposed framework demonstrates the feasibility of integrating large-scale, multi-source health data for cardiovascular risk prediction. Such an approach could be further adapted for integration within Clinical Decision Support Systems (CDSS) to assist healthcare professionals in early cardiovascular risk screening and personalized intervention planning. By integrating automated prediction models with electronic health record platforms, CDSS tools could dynamically assess patient risk in real time, providing data-driven guidance to clinicians while maintaining interpretability and transparency. Future work will focus on external validation with additional clinical datasets and exploring model deployment within CDSS infrastructures to bridge the gap between data-driven research and clinical application. The extensive grid search across multiple ensemble and boosting frameworks required substantial computational resources and time. Training and optimization were performed on high-performance hardware, as the large dataset size and parameter complexity of models such as CatBoost, LightGBM, and XGBoost imposed notable memory and processing demands. These computational challenges emphasize the need for efficient model selection strategies and scalable implementations in future research.

## 5 Conclusion

In this study, we merged two large publicly available health datasets to inspire future research addressing the challenge of finding substantial medical datasets, particularly in the health industry. Using an extensive dataset of 323,681 medical records, we applied innovative machine learning algorithms to predict CVD effectively. By utilizing diverse machine learning models and employing SMOTE to address data imbalance, we demonstrated significant improvements in prediction accuracy. Our findings suggest that leveraging large and varied datasets may enhance the robustness and consistency of machine learning models within integrated datasets in CVD diagnosis.

To further improve early identification and diagnosis of cardiovascular diseases using machine learning, future research should focus on several key areas. First, integrating continuous patient information with real-time health monitoring data could yield more dynamic and timely predictions. Second, exploring deeper learning architectures and advanced ensemble techniques may enhance prediction accuracy and consistency. Additionally, as the use of comprehensive healthcare records expands, addressing data privacy and security concerns will become increasingly critical. Finally, collaborating with healthcare professionals to develop user-friendly decision support systems can facilitate the clinical deployment of these models, ultimately improving patient outcomes and healthcare delivery.

## Data Availability

The original contributions presented in the study are included in the article/Supplementary material, further inquiries can be directed to the corresponding author.
