# Supplementary material for: Machine learning techniques for improved prediction of cardiovascular diseases using integrated healthcare data
Source: Front Artif Intell. 2025 Dec 9;8:1694450. doi: 10.3389/frai.2025.1694450 (PMC12723862; doi:10.3389/frai.2025.1694450)
Supplement: Supplementary file 1 [file Supplementary_file_1.pdf]

Appendix :

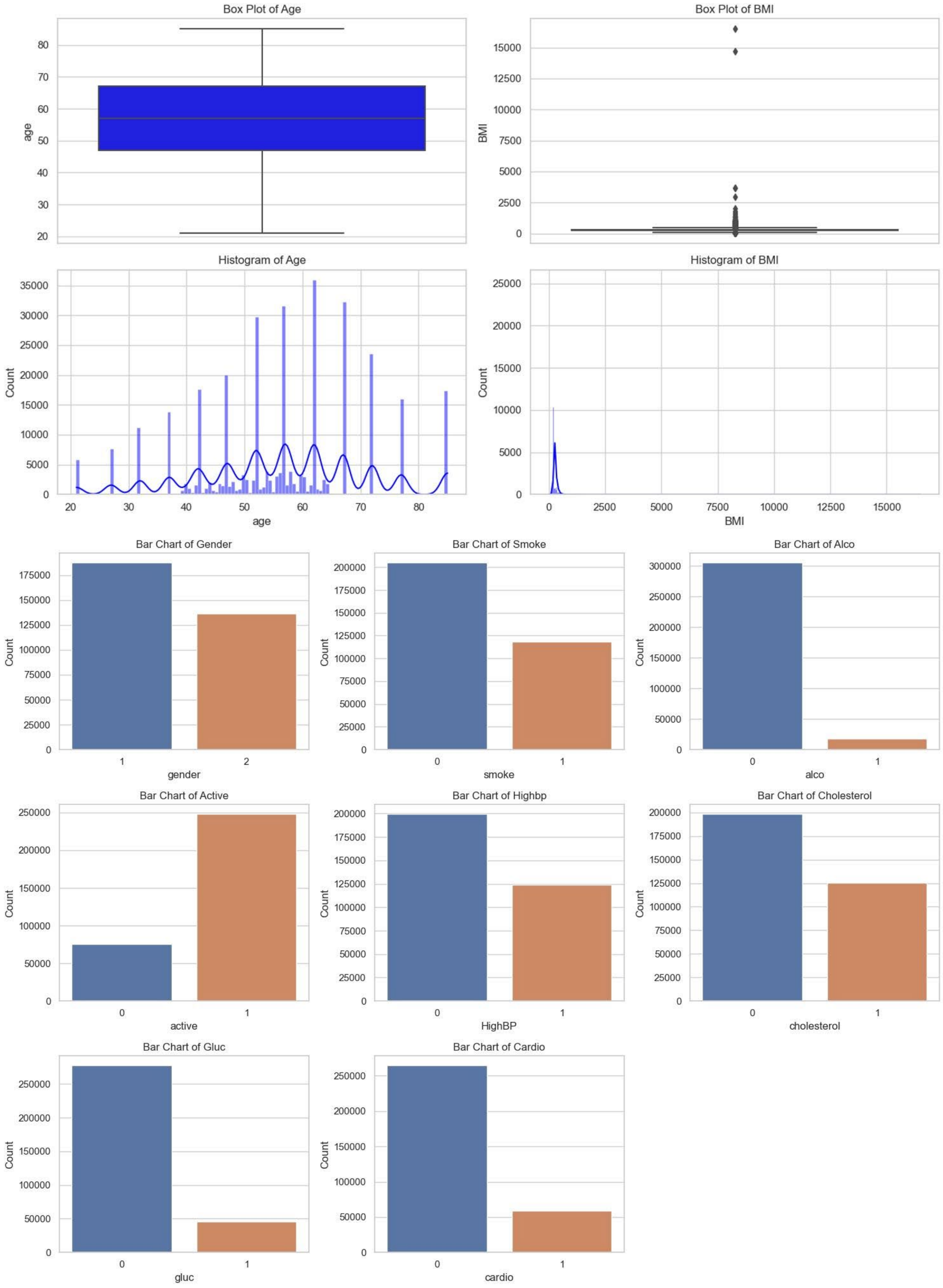

Supplementary Figure 1. Summary statistics and distributions of all input features in the merged dataset, visualized with boxplots, histograms, and bar charts. Highlights include the prevalence of outliers in BMI and the initial class imbalance in cardiovascular disease status.

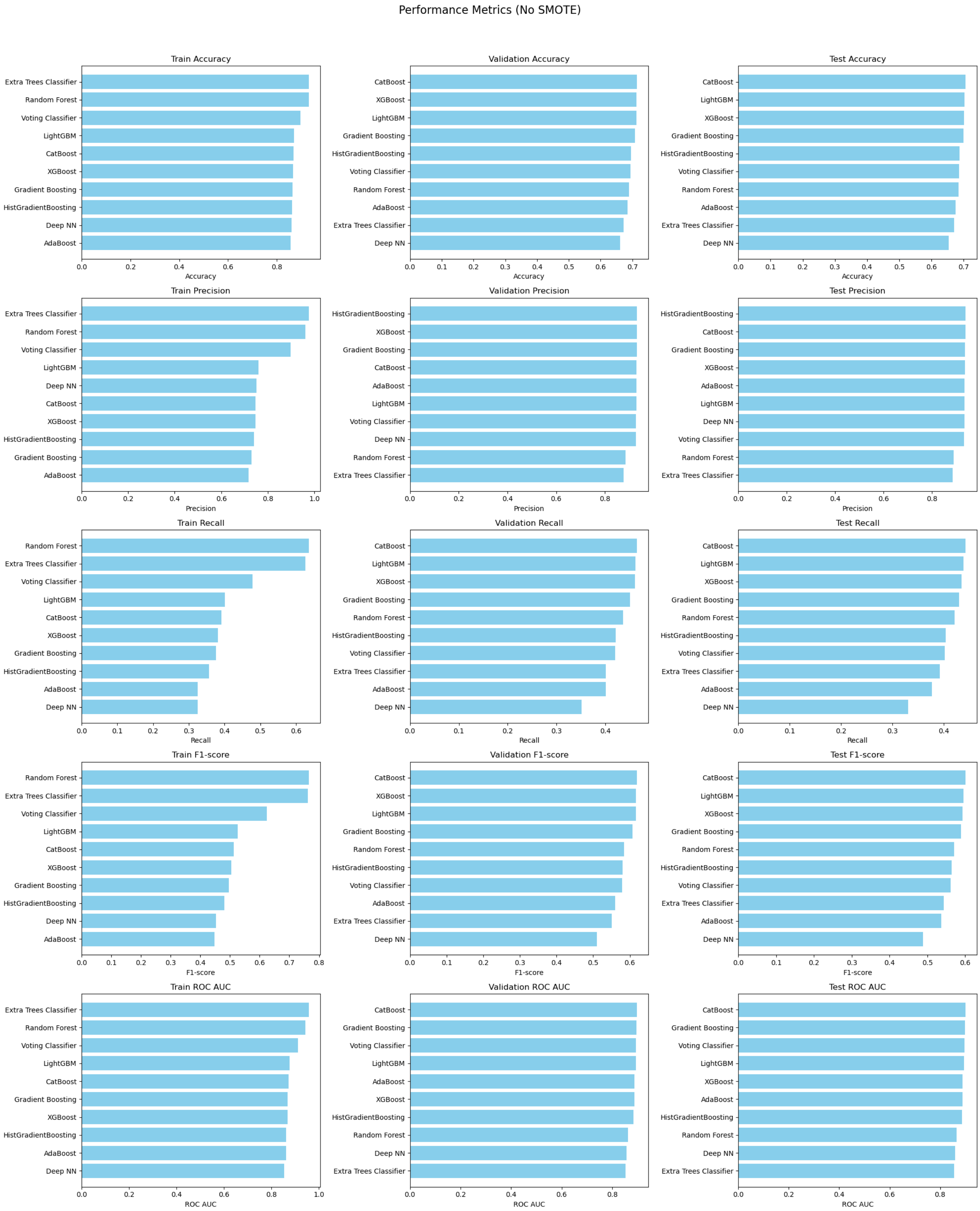

Supplementary Figure 2. Comparative performance of ten machine-learning classifiers before SMOTE application in the training data, based on Accuracy, Precision, Recall, F1-score, and ROC AUC for the training, validation, and test sets. Boosting-based models (CatBoost, LightGBM, XGBoost) exhibit the best baseline results, while individual estimators show lower and less stable performance.

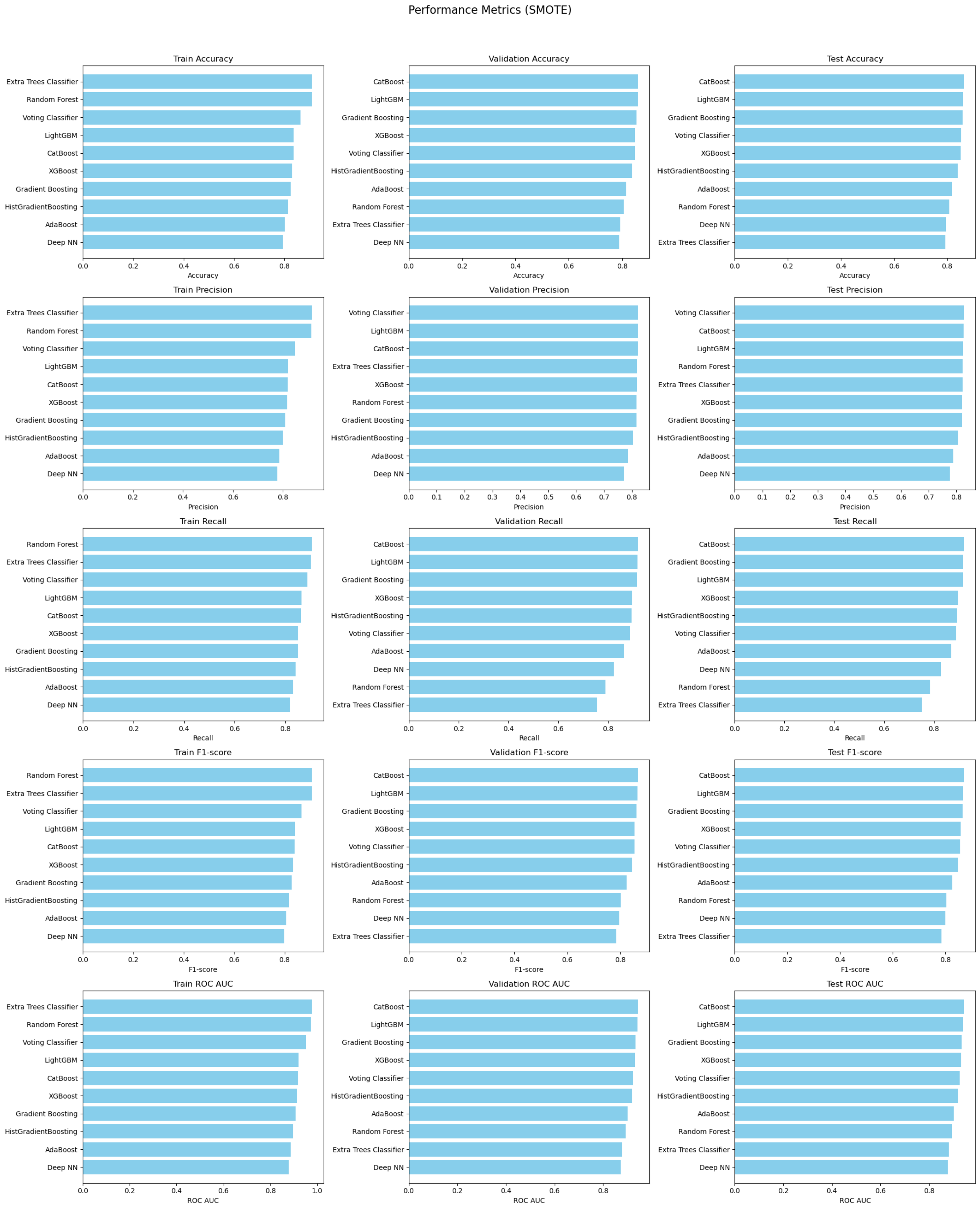

Supplementary Figure 3. Comparative performance of ten machine-learning classifiers after SMOTE resampling in the training data. All models show improved metrics—especially Recall and F1-score—with CatBoost and LightGBM maintaining the highest and most balanced overall performance, demonstrating the benefit of addressing class imbalance.

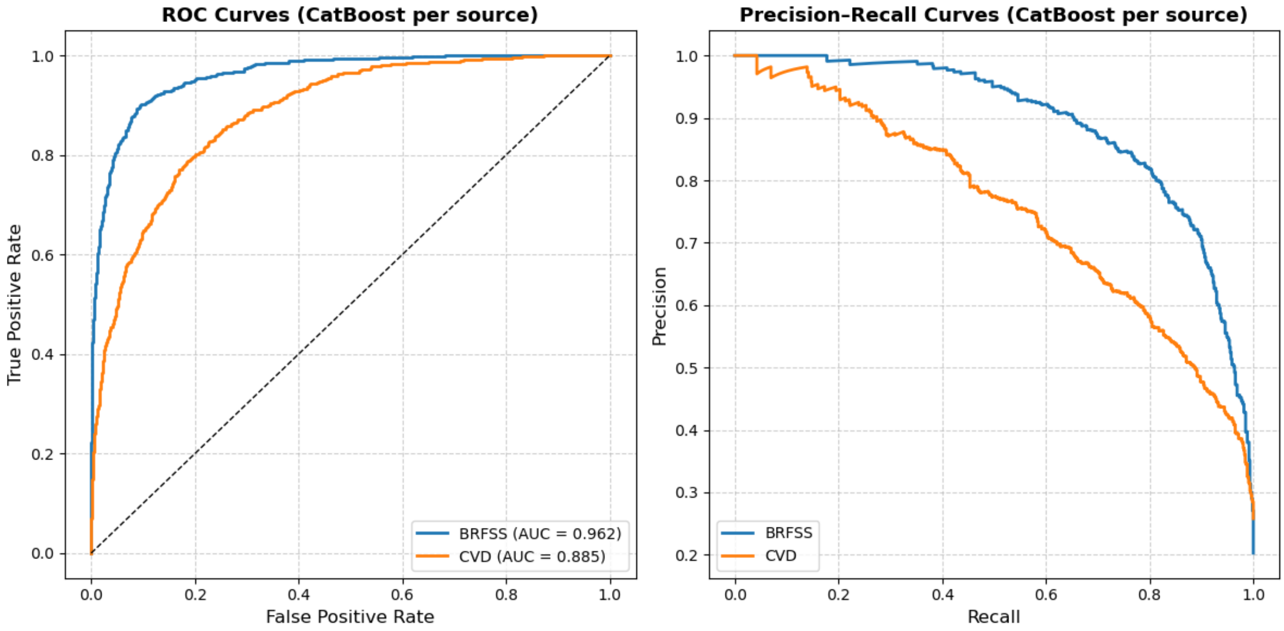

Supplementary Figure 4. Receiver Operating Characteristic (ROC, left) and Precision–Recall (PR, right) curves for the BRFSS and CVD datasets using the CatBoost model trained on SMOTE-balanced training data and evaluated on untouched test sets. Both sources demonstrate realistic and consistent discrimination performance (AUC ≈ 0.89–0.96), confirming that SMOTE was applied exclusively to the training folds without affecting evaluation integrity.

Supplementary Table 1: Supplementary Table 1 presents calibration slope, intercept, and Brier scores for the top-performing models, confirming alignment between predicted and observed outcomes. Summary for the calibration slope, intercept, and Brier scores for top-performing models.

Model	Calibration Slope	Intercept	Brier Score
CatBoost	0.97	0.01	0.084
LightGBM	0.94	0.02	0.091
Gradient Boosting	0.92	0.03	0.096

Supplementary Table 2: Performance metrics with 95% confidence intervals

Model	AUC (95% CI)	F1 (95% CI)	Precision (95% CI)	Recall (95% CI)
CatBoost	0.944 (0.940–0.948)	0.872 (0.868–0.876)	0.827 (0.823–0.831)	0.921 (0.917–0.925)
LightGBM	0.934 (0.929–0.938)	0.864 (0.860–0.868)	0.819 (0.815–0.823)	0.908 (0.904–0.912)
Gradient Boosting	0.912 (0.906–0.917)	0.847 (0.843–0.852)	0.798 (0.794–0.802)	0.893 (0.889–0.897)
